# Changes in Trap Temperature as a Method to Determine Timing of Capture of Small Mammals

**DOI:** 10.1371/journal.pone.0165710

**Published:** 2016-10-28

**Authors:** John L. Orrock, Brian M. Connolly

**Affiliations:** Department of Zoology, University of Wisconsin, Madison, Wisconsin, United States of America; US Geological Survey, UNITED STATES

## Abstract

Patterns of animal activity provide important insight into hypotheses in animal behavior, physiological ecology, behavioral ecology, as well as population and community ecology. Understanding patterns of animal activity in field settings is often complicated by the need for expensive equipment and time-intensive methods that limit data collection. Because animals must be active to be detected, the timing of detection (e.g., the timing of capture) may be a useful proxy for estimation of activity time. In this paper, we describe a new method for determining timing of capture for small mammals. In our method, two small temperature loggers are positioned in each trap so that one logger registers the internal temperature of a live-trap at set intervals while the other logger simultaneously records external trap temperature. We illustrate the utility of this technique using field data from live-trapping of deer mice, *Peromyscus maniculatus*, one of the most ubiquitous, widely distributed small mammals in North America. Traps with animals inside registered consistent increases in internal trap temperature, creating a clear, characteristic temperature deviation between the two data loggers that can determine trap entry time within a very narrow time window (e.g., 10 minutes). We also present pilot data to demonstrate the usefulness of the method for two other small-mammal species. This new method is relatively inexpensive, robust to field conditions, and does not require modification of traps or wiring of new devices. It can be deployed as part of common live-trapping methods, making it possible to assay the timing of capture for a large number of animals in many different ecological contexts. In addition to quantifying timing of capture, this approach may also collect meaningful temperature data and provide insight into the thermal costs of animal activity and relationships between environmental conditions and the time of an animal’s capture.

## Introduction

Small mammals are ubiquitous components of terrestrial food webs that can play many important roles. Small mammals can affect plant population growth and community composition [1], influence the dynamics of other animals (e.g., abundance of arthropod prey, abundance of carnivores; [1]) and serve as important reservoirs for zoonotic diseases [1, 2, 3]. The time when an animal chooses to be in the environment beyond the nest (i.e., activity time) is an important component of small-mammal behavior, ecology, and evolution, since it plays a key role in determining the likelihood of encountering food, stressful microclimates, competitors, mates, predators, parasites, and disease [4, 5]. Various techniques have been used to quantify the timing of small animal activity in the field, including radio telemetry [6], passive monitoring via PIT tags [7] or IR-triggered monitors [8], or by recording the time animals are photographed [9, 10]. Although these methods can be highly informative (see discussion in [8, 11]) many of these methods require expensive, fragile, and/or cumbersome equipment that can be difficult to deploy and maintain under field conditions and may require specialized training to operate. These logistical constraints can limit the number of animals that can be measured in a given sampling period (e.g., the number of animals that can be followed for telemetry). Furthermore, it may be difficult to identify individuals or species using some methods for assessing activity timing (e.g., it may be difficult to identify individuals or species using photos from field cameras), making it difficult to study how activity timing might be affected by individual-level factors (e.g., gender, mass) or how it might differ between species that have a similar appearance [11]. For example, white-footed mice (*Peromyscus leucopus*) and deer mice (*P*. *maniculatus*) may be found in the same habitats but may require detailed inspection, tissue samples, and/or morphological measurements to identify [12].

Because an animal must be active to be captured in a trap, periods of animal activity may also be approximated by using the time that an animal is captured in a live trap [5, 13–16], given the important assumption that the onset of animal activity and the time of capture may not always be correlated [11] (see Discussion for additional details). Quantifying activity time using the time of trap entry may be informative because it is possible to assign activity to a particular individual and species [11], as well as monitor a large number of animals in a single trapping session [13]. If simple methods were available, trap-entry time estimation could be incorporated into live-trapping protocols that are commonly used to monitor animal populations and communities, permitting efficient collection of concurrent data on timing, survival, and abundance [13–15]. Although trap timing has the potential to be informative and can be implemented in any small-mammal live-trapping study, visiting trapping grids many times in a night to manually record capture timing may be time-consuming, may have limited temporal precision, and may have the undesirable consequence of altering animal activity patterns by disturbing habitat [11, 13]. Automated methods for recording the time that an animal enters a trap would eliminate these issues, but automated trap timers are not often commercially available [14] and constructing trap timers from existing electrical components [13, 15, 17], may only work for larger types of trap [17], and may be prohibitive in terms of cost, setup/processing time, and personnel experience.

**Fig 1:**
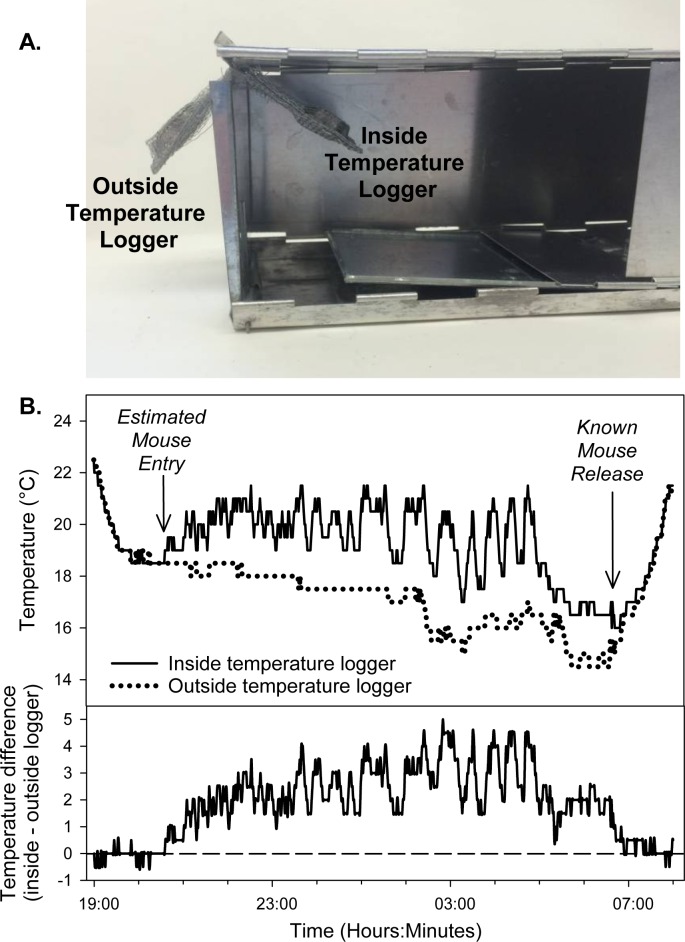
Overview of the method and demonstration of thermal patterns indicative of animal capture. A. Overview of the trap-timing design: paired pouches made from aluminum window screening are used to suspend one temperature logger inside the Sherman live trap and another temperature logger outside the trap. The side of the live trap is opened to provide a clear view of the setup. B. An example of how temperature data can be used to estimate entry time. Temperature differences between the inside and outside of a trap for times when the time of *P*. *maniculatus* release is precisely known. Note how environmental temperature fluctuations affect the temperature inside and outside the trap, but the difference between the paired temperature loggers is a consistent indicator of trap status.

In this paper, we describe a simple method to determine the time that a small mammal is captured in a live trap (Fig 1A). Our method takes advantage of the heat produced by endothermic animals to determine the timing of trap occupancy: if air temperature is measured inside and outside of a trap using small temperature loggers (Fig 1B), heat produced by a captured endothermic animal should cause a predictable, directional shift in the difference between the inside and outside trap temperatures, i.e., the inside temperature should increase while the outside temperature should track ambient conditions (Fig 1B). This technique is simple to implement during live-trapping sessions: it uses data loggers that are widely available and materials that can be purchased in any hardware store. This trap-timing method does not require heavy equipment (e.g., marine batteries) or expensive sensors, and there is no need to drill or otherwise permanently modify the live traps. We present our method using a model of live trap (Sherman Live Traps, Tallahassee, FL) that is among the most commonly used trap in studies describing small-mammal population and community structure [18]. Importantly, our technique is likely to be applicable to any type of small-mammal trap (or nest box) where the animal is housed in a closed area, e.g., the Longworth trap [18]. Moreover, because animal activity patterns are often a function of environmental temperature, this approach also captures additional temperature data that are themselves useful for addressing hypotheses pertaining to behavior, metabolism, and energetics. For example, ambient temperature data can be used to understand how microclimate affects animal activity timing and capture probability, and differences between internal and external temperature loggers may inform questions regarding metabolic demands and thermogenic capacity in different environmental conditions.

## Methods

### Using temperature variation to determine activity time

The protocol used in this research was approved by the UW-Madison Research Animal Resource Center (RARC). Our method of quantifying trap-entry time requires three components for each live trap: two iButton® Thermochron data loggers (Maxim Integrated Inc., San Jose, CA) and a pouch that can be used to hang the data loggers so that one logger is suspended inside the live trap and the other logger is outside the live trap (Fig 1). We utilized loggers with a 0.5°C temperature resolution, set to take a reading every minute; the current cost of each logger is approximately 28–38 U.S. dollars. Initiation of temperature sampling programs and data collection at the end of each trapping session took less than a minute per trap. Pouches used to hold the loggers were constructed by hand with aluminum window screening, with the folded edges stapled together using a standard desk stapler; constructing each reusable pouch took less than 4 minutes and required no special tools or training. Aluminum mesh is an ideal material to house the temperature loggers, as it is readily available at hardware stores, it resists chewing by small mammals, it can be washed and sterilized between each use, and its relatively high thermal conductivity and air flow through the screen allows the loggers to readily integrate and record proximate changes in air temperature. The paired deployment design keeps the two loggers in close spatial proximity, so that small-scale changes in microclimate that are not of interest (i.e., those not caused by small mammals) are likely to be recorded concurrently by both loggers and hence are unlikely to affect the ability to detect small mammal trap entry (Fig 1B). Although we did not evaluate other types of temperature logger, our general paired approach is likely to work with any temperature logger that can be suspended or mounted in close proximity both inside and outside the trap.

Because the assessment of trap entry depends upon temperature changes between the loggers (Fig 1), it is essential to first determine the degree to which the species of interest and planned trapping protocol influence changes in trap temperature. For example, the magnitude of the difference in temperature between the inside and outside of the trap will likely vary based upon the thermal properties of the species captured and ambient temperature, as well as be affected by thermal conductivity between the animal and the interior of the trap and between the trap and the outside environment. These thermal properties will likely differ depending upon the type of trap used (e.g., large or small trap, aluminum or galvanized steel construction) as well as depend upon the presence of insulating material around the outside of a trap, the presence of nesting material (e.g., cotton) inside the trap, and so on. As such, implementing this technique will be most effective when field data are first used to generate an empirical estimation of the expected temperature change for the species likely to be captured in a given study.

**Fig 2:**
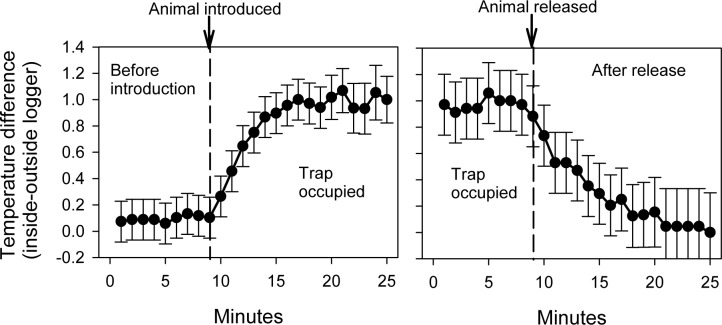
Changes in trap temperature when entry and exit times are known. Temperature changes (delta C) between the inside and outside of a trap for times when the time of *P*. *maniculatus* entry (left panel) or exit (right panel) is precisely known. Data for known-entry trials consist of 45 separate trials conducted over a range of ambient temperatures of 14–20.5 C (mean = 17.09) using 34 different mice ranging in weight from 14.5–27 grams (mean = 19.04). Data for known-exit trials consist of 17 separate trials conducted over a range of ambient temperatures of 14–20.5 C (mean = 18.02) using 17 different mice ranging in weight from 15–24 g (mean = 18.74). Core body temperature of *P*. *maniculatus* is 37 C [19]. Error bars represent 95% confidence limits.

**Fig 3:**
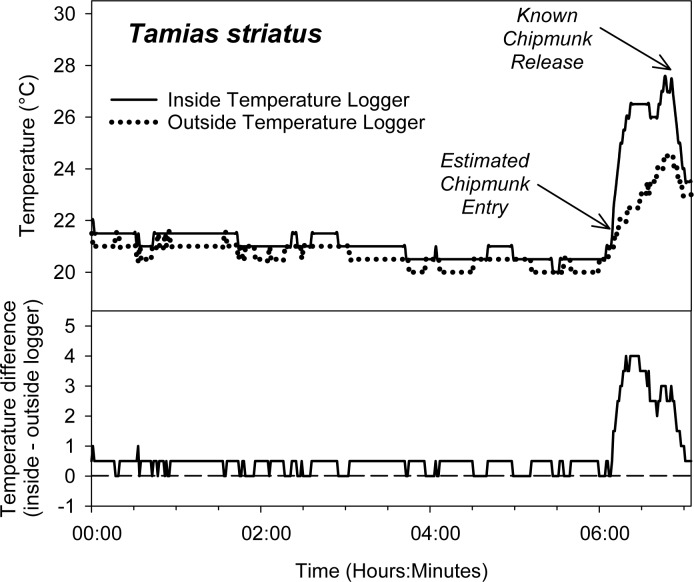
An example of changes in trap temperature following capture of an individual *Tamias striatus*. Trial was conducted in Madison, Wisconsin (latitude 43.096629, longitude -89.331993) on July 24–25, 2015. A live trap was placed in an open, mixed-deciduous forest stand characterized by *Fraxinus* sp. and *Acer* sp. in the canopy, with occasional *Betula papyrifera* and *Picea glauca*. The predominant forb in the understory was an *Arctium* sp. and there were intermittent, shrubby *Morus* sp. present. Weather conditions during the night of trapping were clear and without precipitation. Core body temperature of *T*. *striatus* is approximately 38 C [20].

**Fig 4:**
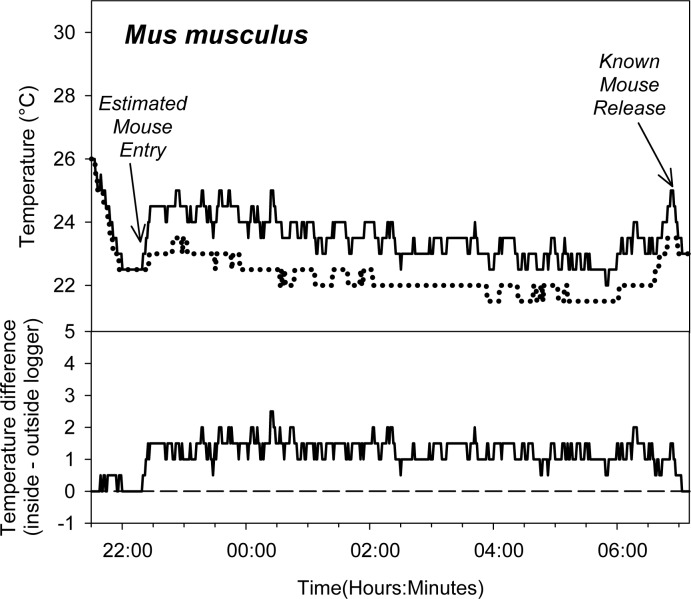
An example of changes in trap temperature following capture of an individual *Mus musculus*. Trial was conducted in Madison, Wisconsin (latitude 43.096629, longitude -89.331993) on July 24–25, 2015. See Fig 3 for a description of sampled habitat and weather conditions. Core body temperature of *M*. *musculus* is approximately 36 C [21].

Two general approaches can be used to estimate the change in temperature that will occur inside the trap once an animal is captured: 1) introducing animals into logger-containing traps at known times, or 2) releasing animals from logger-containing traps at a known time. Both methods provide an estimate of the magnitude of the temperature difference between the inside and outside of the trap created by the animal, and hence can be used to estimate the expected change in temperature when a certain small-mammal species enters a trap and the timing is not known. The latter approach (measuring the change in the temperature difference following a known release event) has the benefit of only requiring one additional, simple step in the field, i.e., recording the time when an animal is released from a trap. However, this latter approach assumes that the rate of trap cooling upon animal release is similar to the rate of trap warming once an animal is captured, and also assumes that the animal has resided in the trap long enough to generate a representative change in temperature (based on our field trials, this time period is relatively short, i.e., less than 15 minutes; Figs 2–4). For this reason, the first method (animal entry into a trap at known time) may provide a more comprehensive estimate of the rate of temperature change following capture as well as the magnitude of the expected temperature shift that will occur following animal entry, assuming that the trials are conducted in a manner that is as similar to the planned field sampling as possible.

**Fig 5:**
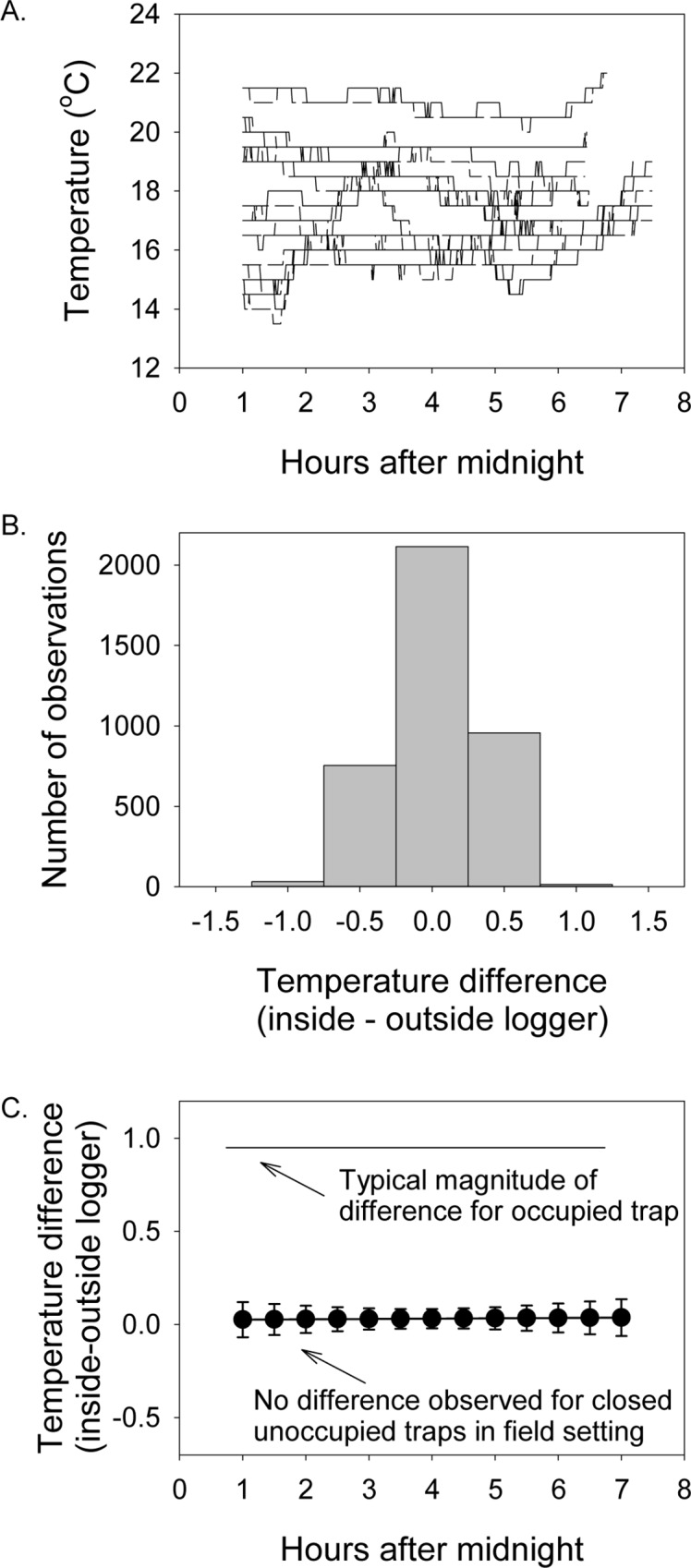
Field-deployed traps with closed doors demonstrate how closely paired loggers track environmental conditions when a trap is not occupied. Trials were conducted in California and Wisconsin field sites (see text for location and habitat information) on July 30–31, August 18, and August 25, 2015 (California sites) and July 24–25, 2015 (Wisconsin). A. Presents raw data from paired loggers inside traps (dashed lines) and outside traps (solid lines). B. A histogram illustrating the very small deviation in temperature between the paired loggers; in 3873 observations, only 14 observations (0.3%) were of the 1-degree magnitude temperature shift expected by animal entry, and none of the deviations were consistent (so none would be mistaken for animal entry). C. Mean values for the temperature difference between inside and outside loggers demonstrates that, throughout the night, the signal due to environmental variation was easily distinguished from the signal expected when an animal was in the trap. Error bars represent 95% confidence intervals.

These known-entry trials and known-exit trials can also be paired with traps deployed in the field that are not capable of capturing animals (e.g., a trap with the door closed so animals cannot enter). Traps deployed in this way provide further evidence of the degree to which environmental temperature variation that is not of interest (i.e., temperature changes not caused by a capture) are present under field conditions. Traps could also be deployed with the trap door locked open (instead of the door closed); however, animals that enter the trap during the night could lead to temperature differences that are not driven by the environment. As such, for the estimation of the role of environmental temperature, we chose to use traps with closed doors. In our experience estimating the capture time for largely nocturnal animals, temperatures inside and outside the trap both track the environment, so that there is no difference for traps where animals are not captured (Fig 5).

Once the magnitude of the change in trap temperature following animal capture is estimated using known entry and/or exit times, trap-entry time for traps where entry time is unknown can be determined by examining the paired trap temperature data (Fig 2). Our field data suggest that, for *P*. *maniculatus*, this method can be used to resolve trap-entry times with high levels of temporal precision (i.e., within 5 minutes, see below). Although the patterns are typically very clear in our experience, determination of trap-entry time via simple examination of the temperature data will necessarily involve some subjectivity. Several statistical approaches can be implemented to make a more formal rule for assessing the timing of capture based upon changes in temperature, including generalized linear models (e.g., logistic regression), generalized additive models, as well as statistical approaches for detecting state transitions in time series [22]. Simple decision rules (e.g., a trap has been entered when there are 3 successive observations where the difference in temperatures is at least 1 degree) may also be useful, as we illustrate below.

### An example using field data

We quantified the magnitude and rate of temperature shift observed when deer mice, *Peromyscus maniculatus* were placed in open traps and released at a known time period shortly thereafter. Deer mice occupy a variety of habitats across North America, are often important components of small-mammal communities [23], can play key roles in the context of plant establishment [24] and mediate the dynamics of diseases of significant human concern, e.g., Lyme disease [25] and Sin Nombre Virus [3]. Deer mice are also useful for testing the efficacy of our method because their body size is similar to a wide range of other small mammal species: for example, 179 non-volant species have average body masses within the 15–25 gram range exhibited by deer mice [26]. Our trials were conducted during rodent live-trapping on four of the California Channel Islands: Santa Rosa Island (latitude 34.002821, longitude -120.062764), Santa Cruz Island (34.051375, -119.567464), Santa Barbara Island (33.473853, -119.034957), and East Anacapa Island (34.014907, -119.363282) from July 30, 2015 to August 25, 2015; there was no precipitation on any of the nights when sampling was conducted. Grassland habitats sampled on these islands were generally characterized by grasses such as *Stipa pulchra*, *Bromus diandrus*, *Avena fatua*, and *Bromus hordeaceus*, as well as shrubs, such as *Lupinus chamissonis* and *Leptosyne gigantea*.

To evaluate the expected temperature shift that would occur within an occupied trap, we quantified temperature shifts in the two ways described earlier: by evaluating trap heating when animals were introduced at a known time and by evaluating trap cooling when the animal was released at a known time. Known-entry-time trials were conducted by allowing traps with loggers to remain in the field overnight with doors locked open using a wooden craft stick. Other live traps that were not locked open were baited with rolled oats and placed nearby; traps were not covered and had no internal insulation. Traps were checked early the next morning. If a mouse was captured, the mouse was transferred to the logger-containing trap and the trap door was shut for approximately 16 minutes. Care was taken to ensure that the logger-containing trap was placed back in its original location. Known-exit-time trials were conducted by releasing an animal captured in a trap that already contained the paired-logger apparatus. Upon release, the trap was placed back in its original location and allowed to cool for approximately 16 minutes or more; after the trial, all animals were released unharmed at the site of capture. When possible, known-entry and known-exit trials were conducted using the same animal and trap, i.e., an animal would be introduced into a trap that had been sitting in the field, allowed to remain in the trap, then released from the trap and the trap was allowed to cool in its original location for a sufficient period. Based upon our data, both approaches provide similar estimates of the size of the temperature shift that will be observed upon capture (Fig 2).

To complement the data from known-entry and known-exit trials, we evaluated the role of environmental temperature variation in affecting the temperature inside and outside of traps by deploying traps with closed doors. Other than the closed door (vs. open door), these traps were placed in the same locations as the traps used for known-entry and known-exit trials (i.e., they were deployed on the four Channel Islands as well as in Wisconsin).

To determine the utility of using simple statistical criteria to assign trap-entry status, we calculated running averages a 5-minute time window (based on preliminary analyses, the 5-minute window performed better than 3, 7, 9, or 11 minutes). We used the data from times of known animal introduction, focusing on 16 capture events with at least 283 minutes of pre-occupancy temperatures and 13 minutes of known occupancy. We utilized these data because we wished to test the statistical criteria for trap-state assignment under conditions where there was a reasonable opportunity for false-positive errors (i.e., unoccupied traps sitting in field conditions, for extended periods of time, as would occur in practice). We classified a trap as “occupied” after the first occurrence of a moving average of greater than 0.5 C.

## Results

The known-entry-time trials and known-exit-time-trials reported here occurred in ambient temperatures ranging from 14.0–20.5 C using animals that ranged from 14–24 g. For known-entry-time trials, interior trap temperatures rapidly diverged once an animal was introduced to the trap, reaching an asymptote within 10 minutes of animal introduction (Fig 2). For known-exit-time trials, the difference in temperatures dropped at a similar rate upon animal release, reaching an asymptote that did not differ from zero within 10 minutes (Fig 2). To directly compare mean changes in temperature prior to capture, during occupancy, and following release capture, we used 1819 minutes of data from 26 trials where both known entry and known exit times were available for the same trap and same captured animal. Ambient temperatures during these trials ranged from 12–31 C, and the masses of captured animals ranged from 14.5–27 grams. These data included an average of 29.35 ± 0.17 (*SE*) minutes of observations for the period immediately prior to occupancy, 23.31 ± 2.98 minutes of observations during occupancy, and 17.31 ± 1.95 minutes of observations following release. The change in the difference between the inside and outside logger was highly significant (*F*_2,49_ = 10.75, *P* < 0.001) as evaluated using a general linear mixed model with a first-order autoregressive covariance structure to accommodate the covariance of measures taken at successive time intervals on the same trap. Linear contrasts indicate that, as expected, the temperature difference was significantly higher when the trap was occupied compared to the period prior to occupancy (*t* = 4.57, 49 *d*.*f*., *P* < 0.001) as well as compared to the period following animal release (*t* = 2.94, 49 *d*.*f*., *P* = 0.003). There was no difference in temperatures between the period prior to occupancy and the period following animal release (*t* = 1.36, 49 *d*.*f*., *P* = 0.179).

Data from traps with closed doors were collected in ambient temperatures ranging from 13.5–22 C. There was no difference between the inside and outside logger (*F*_1,3862_ = 0.09, *P = 0*.*763*): the mean temperature difference was 0.025 ± 0.083 (Fig 5).

**Fig 6:**
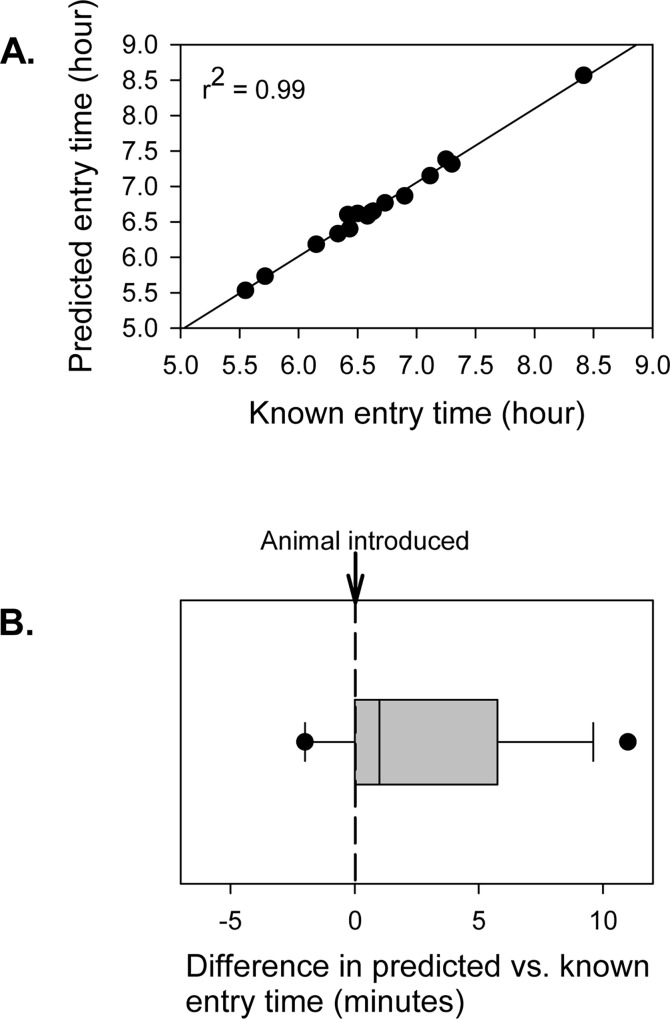
A simple decision rule is effective for predicting trap entry time. A simple decision rule, i.e., the timing of capture is considered to be the first time period when the moving average of the temperature difference over 5 minutes is greater than 0.5, was evaluated using data where the timing of trap entry was known with precision because animals were experimentally introduced.

We used data from known entry times to evaluate the utility of using a simple decision rule to determine when an animal was captured. Based upon the magnitude of temperature change when animals were introduced to traps (e.g., 0.5 degrees or more), we classified a capture as occurring during the first time period when the 5-minute moving average temperature difference was more than 0.5 degrees. This approach performed very well, yielding estimates of trap entry time that were highly correlated with the actual trap entry time (Fig 6; *r*^*2*^ = 0.99, *F*_*1*,*14*_ = 1888, *P* < 0.001) across a wide array of temperatures (14–20.5 C) and initial entry times (Fig 6).

## Discussion

Our method provides a simple method for the estimation of activity time; this method can easily be deployed in conjunction with the live-trapping methods that are commonly used to characterize of small-mammal populations and communities, making it possible to readily assess trap-entry time for a wide range of studies and environments. Our field trials with *P*. *maniculatus* suggest that using changes in trap temperature to assess capture timing is a very effective approach (Fig 1B, Fig 2, Fig 5). Our success with using this method to assess trap-entry timing in deer mice suggests that this approach may be applicable to other small mammals. Nearly 180 non-volant mammal species have average body masses within the 15–25 gram range common for deer mice [26], and the thermal conductance and metabolic rate of deer mice is similar to many small-mammal species [27]. Pilot trap-temperature data from other species corroborates our assertion that this technique is broadly applicable: trap occupancy by an eastern chipmunk (*Tamias striatus*) generated a 4 C temperature difference in less than 10 minutes (Fig 3) and occupancy by a house mouse (*Mus musculus*) generated a 1 C temperature difference between inside and outside data loggers in less than 4 minutes and sustains an average temperature difference of 1.3 C between inside and outside temperature loggers for several hours (Fig 4). Additional trials conducted with *Peromyscus leucopus*, *Myodes gapperi*, and *Blarina brevicauda* (unpublished data) also support the utility of this approach for other species. Because this approach can be easily and systematically implemented across many environments for many species, researchers may be able to develop standardized temperature profiles for specific species related to measurable individual characters (e.g., weight) across ranges of environmental conditions (e.g., ambient temperature) and use these standardizations to directly inform future trap timing estimation.

An additional benefit of our approach is that it generates concurrent environmental and within-trap (i.e., animal influenced) temperature data. Such data, considered independently or paired, could be used to address questions regarding animal physiology in relation to climatic conditions. For example, temperature differences in between inside and outside temperature loggers could be used to describe animal heat production and estimate metabolic demands in adverse climates (e.g., winter). This method would also facilitate studies to examine how environmental temperature affects small mammal activity and probability of capture, as well as possibly permit researchers to estimate differences in metabolic capacity for heat production among species. Our method may also provide insight into how different trap-insulation methods [28, 29] affect the internal temperature of the trap and thus the potential thermal stress experienced by an animal captured in a cold environment. Evaluating how these insulation techniques affect the efficacy of our technique, as well as overall animal stress, are important avenues of future research.

Our technique has several considerations that are important to note. A primary consideration is that researchers using this method will need to collect pilot data with their study species, trapping protocols, and thermal environments of interest prior to full-scale implementation of the approach. This is advised because animal mass, animal insulation, ambient temperature, and trap conductivity may all affect the shift in temperature between the inside and outside of the trap when the animal is captured. By pairing loggers in close proximity, our approach is likely to be robust to local variation in ambient temperature. However, it is still important to deploy traps in a way that ensures that any other factor that could shift trap temperature is minimized. For example, direct solar exposure on the back of a trap would produce a temperature difference between the outside and inside temperature loggers. Although such events would create a different thermal profile than if an animal entered a trap and would thus be distinguishable upon scrutiny of the data (e.g., sunlight on the outside causes a difference in temperature driven by the outside logger, whereas an animal inside a trap causes a difference in temperature driven by the inside logger), they should still be minimized where possible. In the particular case of direct sunlight on traps, this issue is typically addressed as a standard part of capture protocols, as researchers typically cover traps with litter, place traps in consistently shaded locations, or use a shading device to keep direct sunlight from heating the trap and stressing a captured animal.

Another important consideration is that any approach that uses trap-entry timing to estimate activity timing (including ours) is the assumption that the timing of animal capture reflects the timing of the activity of interest [11]. This is an important assumption because the time when an active animal chooses to enter a trap may depend upon the animal (e.g., age, gender, reproductive status), the species, the bait used in the trap, and the environmental conditions when the trap is open. For example, daily weather patterns, the presence of other organisms, and habitat features all affect foraging decisions in small mammals [30, 31], making it possible that these factors also affect the decision of when to enter a trap [11]. The validity of this important assumption could be readily assessed by conducting ancillary trials that couple camera traps with live traps as part of the preliminary sampling described above to determine the shift in temperature expected when the species in the particular sampling area are captured.

Preliminary trials are also likely to be useful for informing other specific details of logger deployment. For example, our method of deployment may provide a means for large animals (e.g., chipmunks, *Tamias striatus*) to exit the trap by pulling on the pouch and opening the rear door of the trap. Two solutions to this issue are to 1) use a small piece of wire to secure the rear door of the trap, rendering it impossible to pull open after loggers have been suspended on either side of the door; or 2) deploy the loggers by hanging them through one of the side vents located on the top of the long metal side of the trap proximate to the back door. Although these solutions are simple to implement, the need to implement them was only apparent because of preliminary sampling with the species of interest.

## Conclusion

Current small mammal live-trapping protocols are the standard for estimating population demography and community composition, as well as for collecting a variety of data on individual small mammals, e.g., morphology, mass, disease status, and parasite load. Our proposed activity timing protocol permits researchers to add new behavioral and environmental axes to their research questions by estimating trap-entry time with a minimal additional amount of financial and time investment. Additionally, fine-resolution measurements of microclimatic temperature variability (i.e., the outside temperature logger) in relation to timing patterns of small mammal activity and estimates of metabolic performance may help estimate how climatic stressors affect performance and behavior of multiple small mammal species. Future studies that evaluate the efficacy of our method in different environments, thermal settings, and species will be essential for demonstrating the usefulness of our approach for informing hypotheses regarding small-mammal behavior and energetics, and how these are affected by the environment.

## Supporting Information

S1 File Known entry data. (CSV)

S2 File Known exit data. (CSV)

S3 File Known entry and exit data. (CSV)

S4 File Closed trap data. (CSV)
